# Management of Glioblastoma Multiforme in a Patient Treated With Ketogenic Metabolic Therapy and Modified Standard of Care: A 24-Month Follow-Up

**DOI:** 10.3389/fnut.2018.00020

**Published:** 2018-03-29

**Authors:** Ahmed M. A. Elsakka, Mohamed Abdel Bary, Eman Abdelzaher, Mostafa Elnaggar, Miriam Kalamian, Purna Mukherjee, Thomas N. Seyfried

**Affiliations:** ^1^Neuro-Metabolism, Faculty of Medicine, University of Alexandria, Alexandria, Egypt; ^2^Neurosurgery, Faculty of Medicine, University of Alexandria, Alexandria, Egypt; ^3^Pathology, Faculty of Medicine, University of Alexandria, Alexandria, Egypt; ^4^Cancer Management and Research Department, Faculty of Medicine, Medical Research Institute, University of Alexandria, Alexandria, Egypt; ^5^Dietary Therapies LLC, Hamilton, MT, United States; ^6^Biology Department, Boston College, Chestnut Hill, MA, United States

**Keywords:** ketogenic diet, Warburg effect, glioblastoma, hyperbaric oxygen, calorie restriction, therapeutic ketosis, chloroquine, epigallocatechin gallate

## Abstract

Few advances have been made in overall survival for glioblastoma multiforme (GBM) in more than 40 years. Here, we report the case of a 38-year-old man who presented with chronic headache, nausea, and vomiting accompanied by left partial motor seizures and upper left limb weakness. Enhanced brain magnetic resonance imaging revealed a solid cystic lesion in the right partial space suggesting GBM. Serum testing revealed vitamin D deficiency and elevated levels of insulin and triglycerides. Prior to subtotal tumor resection and standard of care (SOC), the patient conducted a 72-h water-only fast. Following the fast, the patient initiated a vitamin/mineral-supplemented ketogenic diet (KD) for 21 days that delivered 900 kcal/day. In addition to radiotherapy, temozolomide chemotherapy, and the KD (increased to 1,500 kcal/day at day 22), the patient received metformin (1,000 mg/day), methylfolate (1,000 mg/day), chloroquine phosphate (150 mg/day), epigallocatechin gallate (400 mg/day), and hyperbaric oxygen therapy (HBOT) (60 min/session, 5 sessions/week at 2.5 ATA). The patient also received levetiracetam (1,500 mg/day). No steroid medication was given at any time. Post-surgical histology confirmed the diagnosis of GBM. Reduced invasion of tumor cells and thick-walled hyalinized blood vessels were also seen suggesting a therapeutic benefit of pre-surgical metabolic therapy. After 9 months treatment with the modified SOC and complimentary ketogenic metabolic therapy (KMT), the patient’s body weight was reduced by about 19%. Seizures and left limb weakness resolved. Biomarkers showed reduced blood glucose and elevated levels of urinary ketones with evidence of reduced metabolic activity (choline/N-acetylaspartate ratio) and normalized levels of insulin, triglycerides, and vitamin D. This is the first report of confirmed GBM treated with a modified SOC together with KMT and HBOT, and other targeted metabolic therapies. As rapid regression of GBM is rare following subtotal resection and SOC alone, it is possible that the response observed in this case resulted in part from the modified SOC and other novel treatments. Additional studies are needed to validate the efficacy of KMT administered with alternative approaches that selectively increase oxidative stress in tumor cells while restricting their access to glucose and glutamine. The patient remains in excellent health (Karnofsky Score, 100%) with continued evidence of significant tumor regression.

## Background

Glioblastoma multiforme (GBM) is the most common and malignant of the primary adult brain cancers (1–4). Although survival is better in younger adults (<50 years) than in older adults (>50 years), less than 20% of younger adults generally survive beyond 24 months (3, 5–7). Accumulating evidence from cell culture studies and preclinical models indicate that glucose and glutamine are the primary fuels that drive the rapid growth of most tumors including GBM (8–11). Glucose drives tumor growth through aerobic fermentation (Warburg effect), while glutamine drives tumor growth through glutaminolysis (11–15). The fermentation waste products of these molecules, i.e., lactic acid and succinic acid, respectively, acidify the tumor microenvironment thus contributing further to tumor progression (16–18). Glucose and glutamine metabolism is also responsible for the high antioxidant capacity of the tumor cells thus making them resistant to chemo- and radiotherapies (10, 19). The reliance on glucose and glutamine for tumor cell malignancy comes largely from the documented defects in the number, structure, and function of mitochondria and mitochondrial-associated membranes (10, 20–27). These abnormalities cause the neoplastic GBM cells to rely more heavily on substrate level phosphorylation than on oxidative phosphorylation for energy (28). Hence, the effective management of GBM will require restricted availability of glucose and glutamine.

The current standard of care (SOC) for GBM involves surgical resection and radiotherapy with concomitant and adjuvant temozolomide (TMZ) (3). High dose steroid (dexamethasone) is often prescribed along with the SOC for GBM to reduce vasogenic edema (29–31). It is now recognized that surgical resection and radiotherapy produce significant necrosis and hypoxia in the tumor microenvironment (4, 32). These disturbances disrupt the tightly regulated glutamine–glutamate cycle in the neural parenchyma thus increasing the levels of glutamine and also glutamate, an excitotoxic amino acid that enhances GBM invasion (33–37). Although TMZ increases progression free survival, it has only marginal effect on overall survival and actually increases the number of GBM driver mutations (3, 38). Moreover, dexamethasone not only increases blood glucose levels but also increases glutamine levels through its induction of glutamine synthetase activity (30, 34, 39, 40). The anti-angiogenic drug bevacizumab, which exacerbates radiation-induced necrosis and selects for the most invasive tumor cells, is also widely prescribed to GBM patients (35). Viewed collectively, these observations may help explain why overall survival remains poor for most GBM patients.

Ketogenic metabolic therapy (KMT) is emerging as a viable complimentary or alternative therapeutic strategy for the management of malignant gliomas (35, 41–44). Calorie restriction and low-carbohydrate high-fat ketogenic diets (KD) reduce the glucose needed to drive the Warburg effect while also elevating ketone bodies that cannot be effectively metabolized for energy in tumor cells due to defects in mitochondrial structure and function (23, 27, 42, 45–50). Calorie restriction and restricted KD are anti-angiogenic, anti-inflammatory, anti-invasive, and also kill tumor cells through a proapoptotic mechanism (46, 51–54). Evidence also shows that therapeutic ketosis can act synergistically with several drugs and procedures to enhance cancer management improving both progression free and overall survival (10, 43, 55, 56). For example, hyperbaric oxygen therapy (HBOT) increases oxidative stress on tumor cells especially when used alongside therapies that reduce blood glucose and raise blood ketones (57). Chloroquine inhibition of lysosomal pH can prevent invasive and metastatic tumor cells from obtaining glucose and glutamine through phagocytosis or autophagy (10, 58). The glutamine dehydrogenase inhibitor, epigallocatechin gallate (EGCG) is also proposed to target glutamine metabolism (59). Hence, KMT targets the multiple drivers of rapid glioma growth while enhancing metabolic efficiency in normal brain cells.

### Ethics Statement

This study has been reviewed and approved by the Chair of the faculty of Medicine Alexandria University Medical Research Review Board (metabolic management of GBM patients along with the SOC therapy, protocol number 69/2016). Following IRB-approved directions for this study, a written informed consent was obtained from the participant for the publication of this case report.

## Case Report

**Figure 1:**
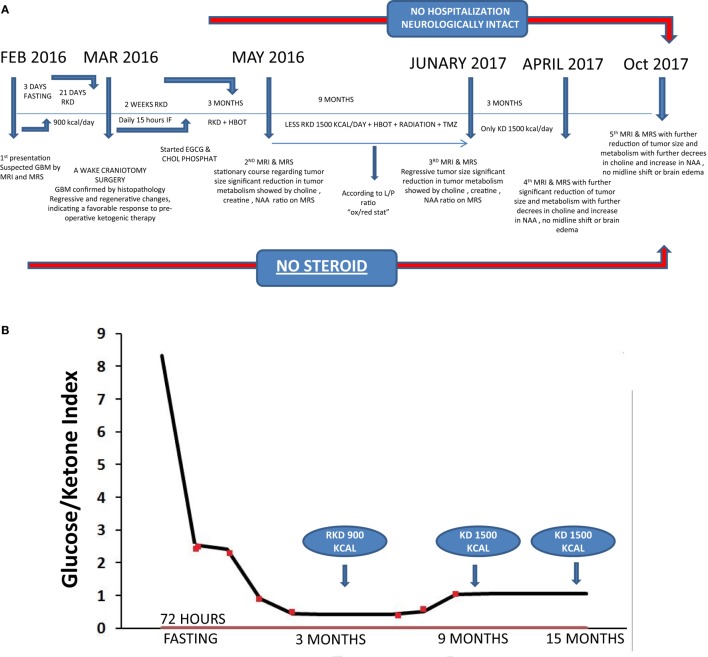
**(A)** Timeline of clinical course with dates of dietary treatments, magnetic resonance imaging (MRI), magnetic resonance spectroscopy (MRS), and hyperbaric oxygen therapy (HBOT). **(B)** Glucose/ketone index indicates the ratio of circulating glucose to urinary ketones at all eight clinical assessments during the 15 months period from February 2016 to April 2017.

**Figure 2:**
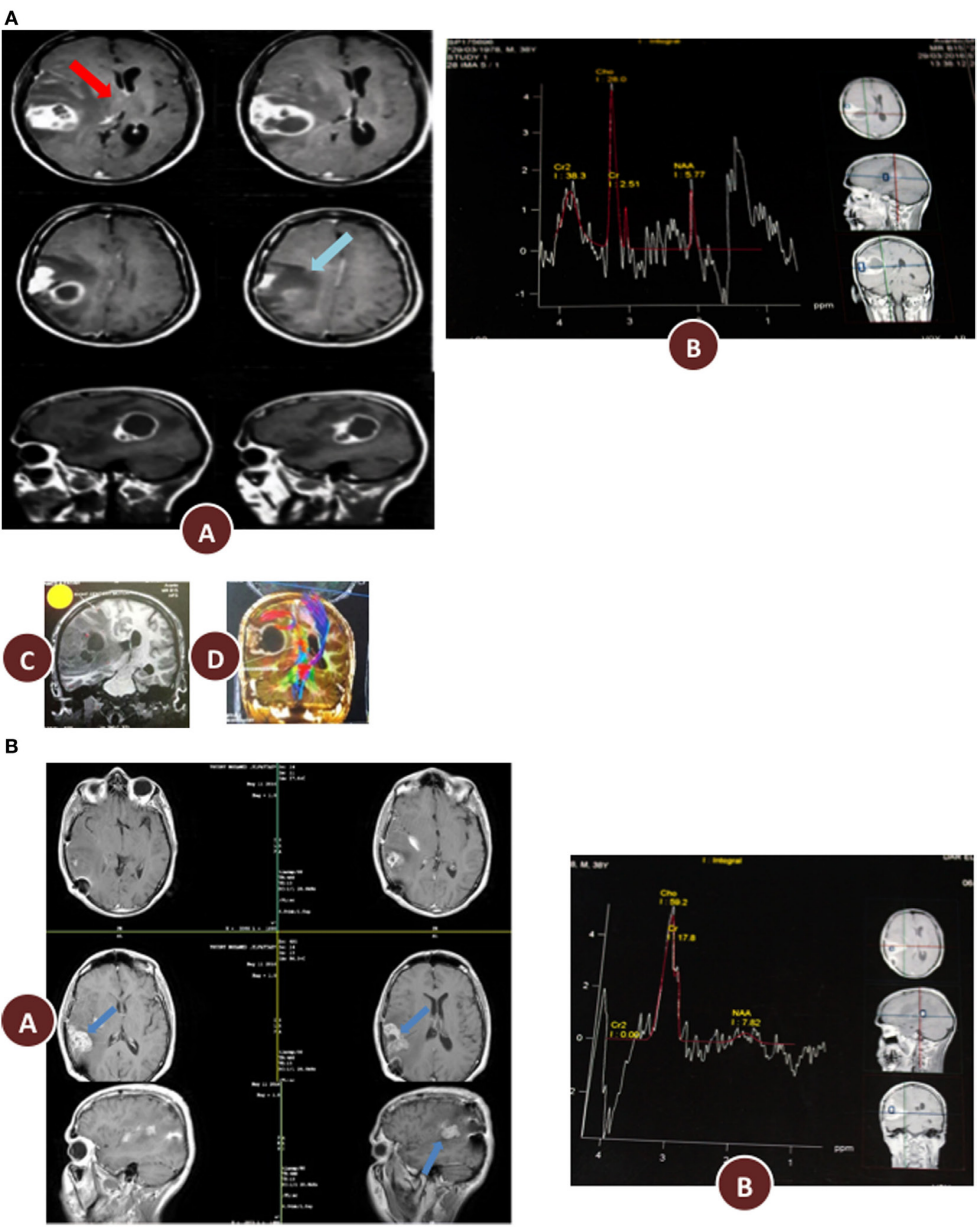

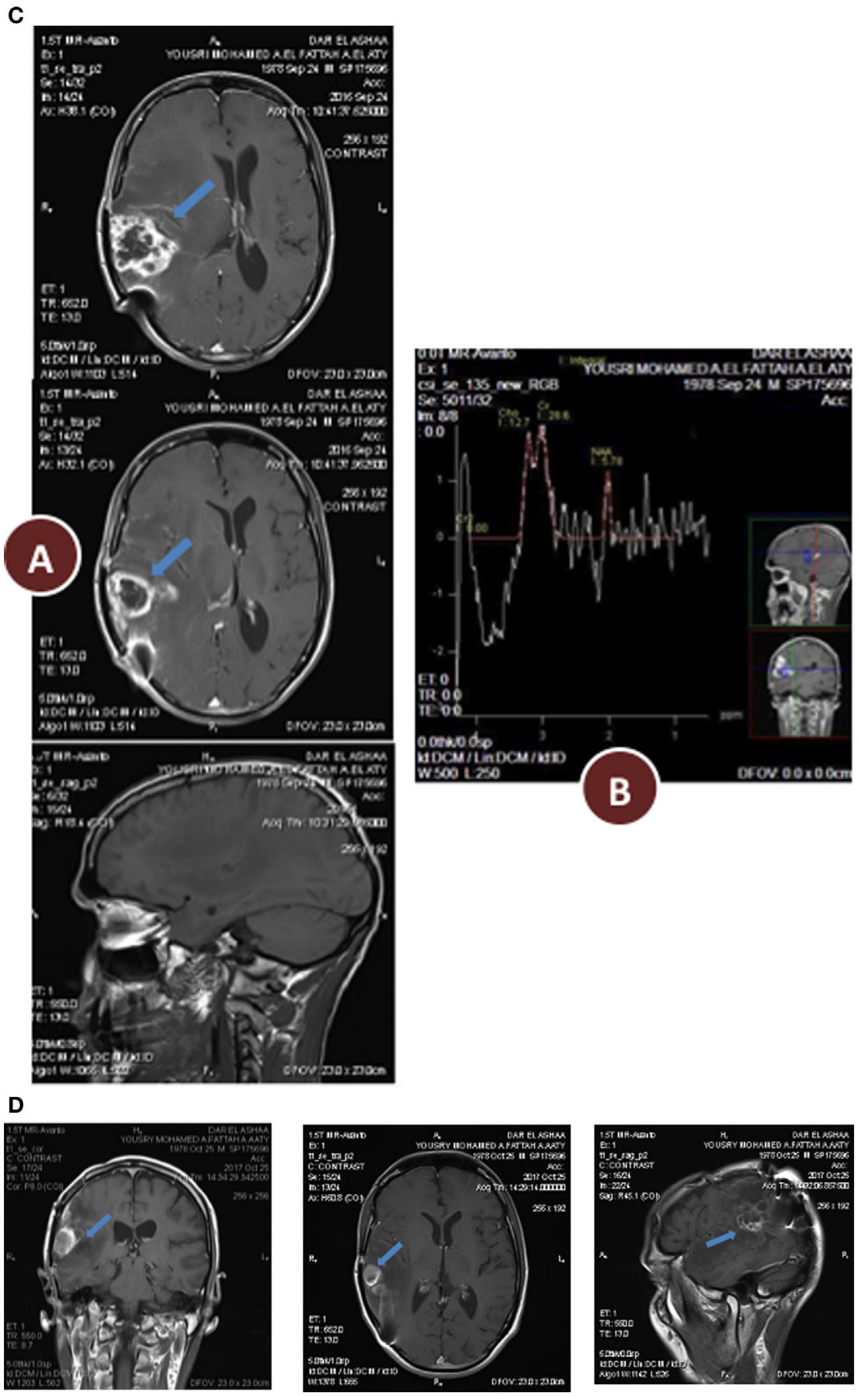
**(A)** Preoperative radiology, (A) magnetic resonance imaging (MRI) contrast enhanced images axial and sagittal view revealed cystic lesion with perifocal edema “blue arrow” and midline shift “red arrow”; (B) magnetic resonance spectroscopy (MRS) of the lesion revealed high choline value (28) and low N-acetylaspartate (NAA) value (2.7); (C) Functional MRI showed affection of motor and sensory functions; (D) MRI tractography showed displaced motor and sensory fibers. **(B)** 3 months postoperative radiology (A) MRI contrast enhanced images axial and sagittal view revealed reduction in tumor size and perifocal edema with less midline shift; (B) MRS of the lesion revealed reduction of choline value (21) and elevation of NAA value (3.5) compare to preoperative study. **(C)** 12 months postoperative radiology (A) MRI contrast enhanced images axial and sagittal view revealed stationary or slightly decrease in tumor size and less perifocal edema with less midline shift; (B) MRS of the lesion revealed reduction of choline value (19) and elevation of NAA value (4) compare to previous study. **(D)** Postoperative radiology at 20 months. MRI contrast enhanced images for axial, sagittal, and coronal views revealed further reduction in tumor size, with no perifocal edema or midline shift.

A 38-year-old male presented on February 2016 with chronic headache, nausea, and vomiting with left partial motor seizures and weakness in the upper left limb (Figure 1A). The symptoms persisted for about 3 weeks before further diagnostic and radiological evaluation. Neurological examination revealed grade 4 left upper limb weakness with mild left facial deviation. There was no history of chronic disorders or malignancy. The patient’s blood pressure was within normal limits (110/70). Laboratory investigation revealed unremarkable blood chemistry, with liver and renal functions within normal limits (Table 1). Blood homocysteine level was elevated, while blood lipid analysis showed hypercholesterolemia and hypobetalipoproteinemia with mildly elevated levels of triglycerides (Table 1). Fasting blood glucose was normal, but fasting insulin level was elevated suggesting some degree of insulin resistance. The patient’s level of circulating 25(OH)D3 was low (3.1 ng/dL). The patient was heterozygous for mutations (c677t and a1298c) in the methylenetetrahydrofolate reductase (MTHFR) gene suggesting a folate deficiency. Enhanced magnetic resonance imaging (MRI) of the brain showed a solid cystic intra-axial occupying lesion in the right partial space (Figure 2A). MR tractography revealed displaced motor and sensory fibers. The preliminary diagnosis was GBM.

**Table 1:** Influence of ketogenic metabolic therapy (KMT) on the patient’s biomarkers.

	Before KMT/first presentation	After KMT/before surgery	KMT continued/after surgery (months)			
Biomarkers			3	9	15	20
Hb (g/dL)	11.0	12.5	13.0	12.9	13.1	16.1
WBC/μL blood	5,000	4,200	3,200	4,500	5,500	4,200
Platelets/μL blood	162,000	171,000	220,000	280,000	310,000	186,000
Cholesterol (mg/dL)	115	–	–	168	170	225
LDL (mg/dL)	50	–	–	103	106	159
HDL (mg/dL)	34	–	–	48	51	62
TG (mg/dL)	155	–	–	83	81	101
25(OH)D3 (ng/dL)	3.1	18.0	32.0	48.0	55.0	42.0
Homocysteine (μM)	19.2	16.0	14.9	12.0	9.4	11.9
Fasting glucose (mg/dL)	89	72	64	75	71	65
Fasting insulin (μIU/mL)	13.10	6.50	5.00	4.10	3.80	2.11
Urine ketones	UD	+++	+++	++	+	++
Weight (kg)	71.1	–	67.7	56.9	66.2	61.8
BMI (kg/m^2^)	25.10	–	23.70	19.90	23.17	21.60

*UD, undetectable*.

The patient’s caloric intake at diagnosis was approximately 2,200–2,500 kcal/day (estimated from a 3-day food record and a 24-h diet recall). The patient underwent a 72 h water-only fast immediately after preliminary diagnosis and before any medical or surgical treatment (Figure 1A). Consumption of a calorie restricted ketogenic diet (KD-R) for 23 days followed the fast. The patient’s biomarker profile before and after fasting and KD-R is shown in Table 1. Measurements of glucose, ketones, and insulin were taken at the laboratory in the morning. Urine ketones were scored as +, ++, and +++ corresponding to median capillary blood ketone levels of 0.5 mmol/L [interquartile range (IQR): 0.1–0.9], 0.7 mmol/L (IQR: 0.2–1.8), and 3 mmol/L (IQR: 1.4–5.2), respectively.

A KD (4:1, fat:protein + carbohydrate) was administered to the patient in restricted amounts for 21 days after the fast and before surgical tumor resection (Figure 1A). This KD-R delivered an average of 900 kcal/day in total, which included: (1) 71 g = 639 kcal fat (33% as olive/flaxseed oils; 33% as medium-chain triglycerides; 33% as organic butter); (2) 50 g = 200 kcal protein (poultry, fish, eggs, with no more than 15% dairy); (3) carbohydrate 15 g = 60 kcal (mainly from green leafy vegetables after subtracting the amount of carbohydrates in protein sources); and (4) 20 g = 0.1 kcal fiber. Meals were designed using the EMK software program. The KD-R was supplemented with B-complex vitamins, minerals, calcium, magnesium, and omega 3 fatty acids to maintain nutrient adequacy and normal blood chemistry. Vitamin D3 was prescribed (5,000 IU/day) to correct the patient’s vitamin D deficiency. Other additions included metformin (1,000 mg/day) and methylfoliate (1,000 mg/day) to overcome MTHFR enzymatic blockage, to correct DNA hypomethylation, and to help decrease homocysteine accumulation. The patient received levetiracetam (1,500 mg/day) to control seizure activity. Immediate preoperative laboratory investigation revealed correction of the previous vitamin D deficiency, a return of homocysteine and triglycerides to normal levels, and correction of hypercholesterolemia and hypobetalipoproteinemia (Table 1). The patient’s glucose-ketone index (GKI), a calculation that tracks the ratio of blood glucose to ketones as a single value, remained in the predicted therapeutic range (Figure 1B) (60). No steroids (dexamethasone), phenytoin, or sugar-based osmatic diuretics (mannitol) were given to the patient.

**Figure 3:**
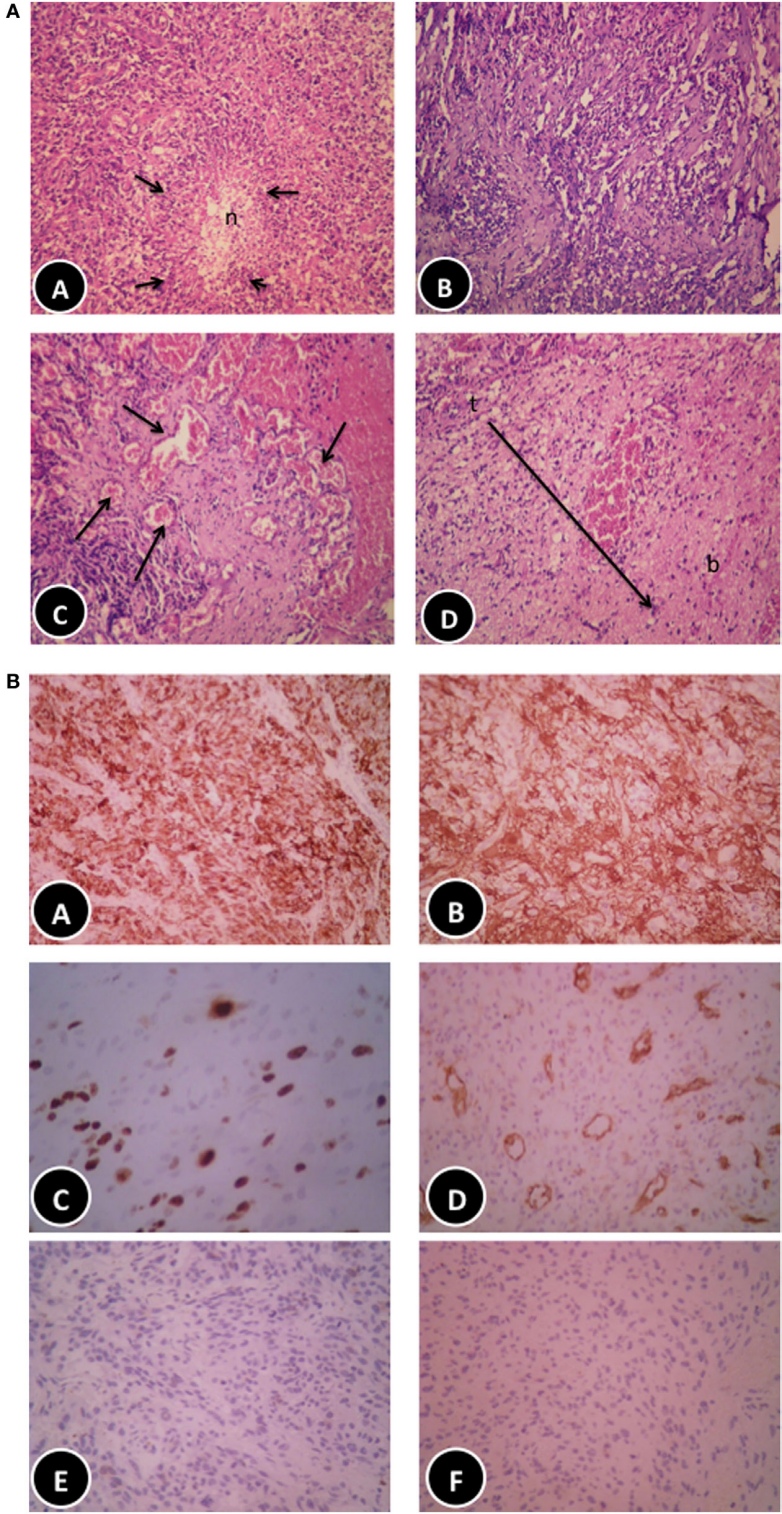
**(A)** Histopathological features of the patient glioblastoma multiforme (GBM). (A) Classic GBM area with pseudopalisading necrosis. (B) Regressive changes in the form of variable background fibrosis. (C) Glomeruloid thick-walled blood vessels. (D) Tumor edge showing limited brain infiltration (haematoxylin and eosin, 200×). **(B)** Immunohistochemical features of the patient GBM. (A,B) Diffuse positive staining for glial fibrillary acidic protein (100× and 200×, respectively). (C) Moderately high proliferative activity (Ki67, 200×). (D) Numerous blood vessels highlighted by cluster of differentiation 31 immunostaining (100×), negative staining for epithelial markers: CK [(E) 200×] and epithelial membrane antigen [(F) 200×], respectively.

After 21 days following initiation of the KD-R, the patient underwent an awake craniotomy with subtotal tumor resection (March 2016). The suspected diagnosis of GBM was confirmed by histopathology of tumor tissue. Regressive and regenerative changes not typical of untreated GBM were noted, possibly reflecting a favorable response to preoperative ketogenic therapy (Figure 3A). Haematoxylin and eosin histological analysis revealed the classical patterns of GBM (Figure 3A). The tumor was composed of a heterogeneous mixture of cells having pleomorphic hyperchromatic nuclei and variable amounts of eosinophilic cytoplasm set in a fibrillary background. Gemistocytic cells (<20%) were focally admixed. Focal epithelioid and clear cell features were also noted. Numerous mitotic figures, vascular proliferation, areas of necrosis (constituting 50% of the tumor), and focal pseudopalisading necrosis were seen. It is noteworthy that less aggressive forms of vascular proliferation and mitosis (namely, glomeruloid vascular proliferation and granular mitoses, respectively) were noted together with the more ominous type of vascular proliferation and irregular mitoses (3/10HPF), more typically associated with GBM (Figure 3A). Also noted was limited infiltration of tumor cells into the surrounding parenchyma. Thick-walled hyalinized blood vessels, often seen in lower grade tumors, were also notable given that this patient had not yet received SOC. Granulation tissue formation and proliferating fibroblasts (organized around areas of necrosis) was another feature not usually present in untreated glioblastomas and suggested a favorable response to preoperative ketogenic therapy. The tumor tissue stained positive for GFA and highly positive for the Ki67 proliferative index and the cluster of differentiation 31 vascularization index, but was mostly negative for epithelial markers (Figure 3B). The immunostaining data supported the diagnosis of glioblastoma and excluded low-grade gliomas and metastatic carcinomas.

The patient’s postoperative recovery was excellent with no signs of previous left weakness or seizures. The patient’s blood glucose level was 64 mg/dL and his urine ketone level was +++, producing an approximate GKI of 1.3. There was no intraoperative or postoperative increase in blood pH assessed by five arterial blood gas measures taken at consecutive hours. The patient was discharged to home 2 days after surgery. The R-KD was continued together with 14 h daily fasting between dinner and breakfast. Together with the previously mentioned medication and supplements, the patient also received chloroquine phosphate (150 mg/kg) and EGCG (400 mg/day). Two weeks postoperative, the patient began receiving HBOT (2.5 ATA for 60 min, 5 times/week). The baseline serum lactate/pyruvate ratio (L/P ratio) test showed an increased lactate ratio indicative of an abnormal oxidation-reduction state.

**Figure 4:**
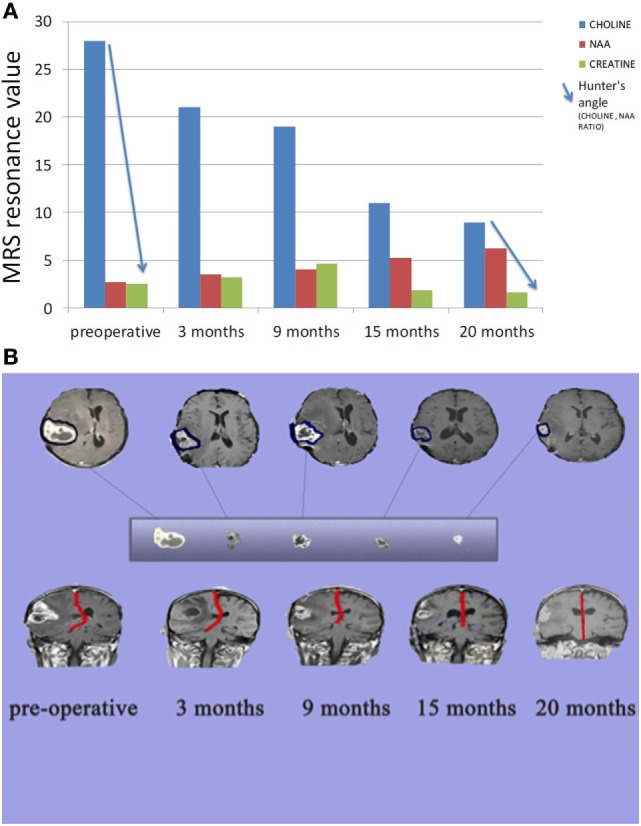
**(A)** Comparison between tumor metabolism over 20 months. *Choline* indicates cell membrane turnover and reflects tumorigenesis. N-acetylaspartate (NAA) is a marker for neuronal integrity that decreases with brain malignancy and radio necrosis. *Creatine* is a marker for cellular energy that decreases significantly with malignancy and radio necrosis. *Hunter angle* (blue arrow) reflects the choline/NAA ratio. **(B)** Comparison between tumor size and midline shift (red line) over 20 months.

Enhanced brain MRI and magnetic resonance spectroscopy (MRS) evaluated 3 months postoperative and before radiation or chemotherapy revealed a stationary course of disease regarding tumor size that persisted to 12 months (Figures 2B,C). The choline creatine ratio on MRS indicated significant reduction in tumor metabolism (Figure 4A). The patient was neurologically intact and free of clinical seizure activity using only levetiracetam (1,500 mg/day) and KD. Fasting glucose was 60–70 mg/dL with ++ to +++ urine ketone levels and producing approximate GKI value of 1.8. Circulating insulin was low (approximately 4 IU) compared with the initial assessment (13 IU). The patient’s body weight was 67.7 kg with a BMI of 23.7. Radiotherapy with oral TMZ (75 mg/m^2^ orally once a day for 42 days) was initiated 18 h after water-only fasting (no food was consumed for up to 8 h following RT and TMZ). The patient also received 20 sessions of HBOT (2.5 ATA, 5 days/week, for 60 min/session). The L/P ratio, known to be elevated in GBM patients, was measured every week and sessions were repeated whenever L/P ratio increased (61). HBOT was also used to help lower the L/P ratio within normal limits (62, 63). The L/P ratio is also a good serum indicator for HBOT effectiveness on tumor oxygenation, glucose metabolism, and tumor redox state.

The patient continued with 30 sessions of brain radiation and completed his TMZ loading without significant side effects or noticeable neurological deficits. After 9 months of therapy, the patient’s weight was reduced to 56.9 kg (BMI 19.9). Despite the reduced BMI, the patient experienced no distress or discomfort. At this point, the patient transitioned to consuming an unrestricted KD with total calories around 1,500 kcal per day. Fasting blood glucose was around 70–75 mg/dL and urine ketone levels were + to ++ producing an approximate GKI of 5.0 (Figure 1B).

After 20 months of metabolic therapy and completion of radio and chemotherapy, the patient’s weight was 66.2 kg (BMI 23.2). Enhanced brain MRI and MRS revealed decrease in tumor size of about 1.5 cm in each diameter, with minimal perfusion and low metabolic activity assessed from the choline to creatine and choline to N-acetylaspartate (NAA) ratios on MRS (Figure 2D). Fasting insulin, glucose, and urine ketones were 2.1 IU, 65 mg/dL, and + to ++, respectively, producing an approximate GKI of 5.0 (Table 1). Also seen were further decreases in choline and increase in NAA with no midline shift or brain edema (Figure 2D), A reduction in tumor size was correlated with a correction of the midline shift after 20 months of treatment (Figure 4B). The patient remains in good health with no noticeable clinical or neurological deficits (Karnofsky Score, 100%).

## Discussion

In this case report, we describe a favorable therapeutic response to KMT and other treatments targeting metabolism in a 38-year-old man with GBM and metabolic imbalances. KMT is a nutritional anti-neoplastic intervention involving ketogenic or low-glycemic diets for managing malignant gliomas (42). The SOC for GBM was modified in this patient to initiate KMT prior to surgical resection, to eliminate steroid medication, and to include HBOT as part of the therapy. Specific drugs and dietary supplements were also used in the therapy. Previous studies showed that a KD is well tolerated for most GBM patients, but therapeutic efficacy can vary among patients perhaps due to differences in the degree of calorie restriction (42, 64–69). Few of the adult GBM patients treated with KDs in these studies were able to reach or maintain the GKI predicted to have the greatest therapeutic benefit for patients (near 1.0) (60). However, an excellent therapeutic response to the KD was seen in two children with high-grade gliomas (70). When compared to baseline, blood glucose levels were lower and blood ketone levels were higher in both children at 8 weeks, with the glucose and ketone values in the theoretical therapeutic zone (60). The observed reduction in blood glucose in our patient would reduce lactic acid fermentation in the tumor cells, while the elevation of ketone bodies would fuel normal cells thus protecting them from hypoglycemia and oxidative stress (10).

Previous studies showed that GBM survival and tumor growth was correlated with blood glucose levels, i.e., the higher was the blood glucose, the shorter was the survival, and the faster was the tumor growth (31, 40, 71–76). As the dexamethasone steroid is often prescribed together with the SOC for GBM (31, 40), the elimination of this steroid treatment could have contributed in part to the reduced glucose levels and favorable outcome observed in our patient. Evidence indicates that glioma cells cannot effectively use ketone bodies for energy due to defects in the number, structure, and function of their mitochondria (10, 23, 27, 42, 45–50, 77). The accuracy of the GKI as a predictor for therapeutic efficacy, however, is better when ketone bodies are measured from the blood than when measured from the urine (10). Nevertheless, the low GKI values observed in our patient following KMT was in the direction of predicted therapeutic success for reducing lactic acid fermentation (41, 60). A reduction of glucose-driven lactic acid fermentation would not only increase tumor cell apoptosis, but would also reduce inflammation and edema in the tumor microenvironment thus reducing tumor cell angiogenesis and invasion (51, 52, 54, 78, 79).

Although KMT is effective in targeting the Warburg effect in GBM cells it would be less effective in targeting glutamine, the other major fuel that drives GBM growth (8, 10, 14, 42). Besides serving as a metabolic fuel for GBM, glutamine is also an essential metabolite for normal immune cells (10, 80). Macrophages in particular are needed to repair the microenvironment and to remove dead tumor cells following metabolic therapy (10). Therefore, therapies that inhibit glutamine availability and utilization must be strategically employed to avoid inadvertent impairment of immune cell functions. It can be difficult to restrict glutamine without also impairing important functions of normal immune cells. Consequently, we used the non-toxic green tea extract, EGCG, and chloroquine in an attempt to limit glutamine availability to the tumor cells. EGCG is thought to target the glutamate dehydrogenase activity that facilitates glutamine metabolism in GBM cells (59, 81). Chloroquine, on the other hand, will inhibit lysosomal digestion thus restricting fermentable amino acids and carbohydrates from phagocytosed materials in the tumor microenvironment (10, 82). We also treated the patient with HBOT to increase oxidative stress in the tumor cells (10, 57). We originally described how somatic mutations could make tumor cells more vulnerable to physiological stress than normal cells based on the evolutionary concepts of Potts (83, 84). As glucose and glutamine fermentation protect tumor cells from oxidative stress, reduced availability of these metabolites under ketosis could enhance the therapeutic action of HBOT, as we recently described (10). The observed reductions in the L/P and choline/creatine ratios coupled with increased NAA are consistent with research suggesting that metabolic management may improve survival (85, 86). We cannot, however, rule out the possibility that our patient’s tumor contained certain genes (IDH1 mutation, ATRX and 1p/19q deletion) that might have also contributed to his favorable response. More detailed studies in other GBM patients surviving to 24 months would be needed to test this hypothesis. We suggest that the targeting of glucose and glutamine in a press-pulse therapeutic strategy together with a modified SOC could have contributed to the favorable outcome in this GBM patient despite evidence of postoperative residual tumor.

## Concluding Remarks

Glioblastoma multiforme remains among the most aggressive and difficult to manage primary tumors of the central nervous system. Emerging evidence indicates that cancer is primarily a mitochondrial metabolic disease where tumor cells become dependent on fermentation for growth. Glucose and glutamine are the prime fermentable fuels that drive GBM cell growth and invasion. A press-pulse therapeutic strategy was implemented to target glucose and glutamine availability in a 38-year-old GBM patient using a modified SOC and KMT. As less than 20% of younger adults generally survive beyond 24 months with GBM, it is possible that the response observed in this case resulted in part from KMT and the modified SOC. The patient is now 40 years old and remains in excellent health with no noticeable neurological issues (Karnofsky Score, 100%) after 24 months of treatment.

## Ethics Statement

This study has been reviewed and approved by the Chair of the faculty of Medicine Alexandria University Medical Research Review Board (metabolic management of GBM patients along with the standard of care therapy, protocol number 69/2016). Following IRB-approved directions for this study, a written informed consent was obtained from the participant for the publication of this case report.

## Author Contributions

AE: conceived the study, collected the data, and wrote the paper. MB: conducted surgical procedures related to standard of care. EA: conducted the pathological report. ME: assisted in data collection. MK: provided information on nutritional status and helped write the paper. PM: evaluated data and assisted in manuscript preparation. TS: helped write the manuscript and assisted in data presentation and analysis.

## Conflict of Interest Statement

MK was employed by Dietary Therapies LLC. All other authors declare that the research was conducted in the absence of any commercial or financial relationships that could be construed as a potential conflict of interest.

## Abbreviations

KD-R, calorie restricted ketogenic diet; GFAP, glial fibrillary acidic protein; KMT, ketogenic metabolic therapy; EMA, epithelial membrane antigen; CK, cytokeratin; CD31, cluster of differentiation 31; Ki67, antigen KI-67 also known as Ki-67 or MKI67 is a protein that in humans is encoded by the MKI67 gene; HBOT, hyperbaric oxygen therapy; MRI, magnetic resonance imaging; MRS, magnetic resonance spectroscopy; EGCG, epigallocatechin gallate; NAA, N-acetylaspartate; L/P, lactate/pyruvate ratio; IQR, interquartile range; dkl, decaliter; EMK, electronic ketogenic manager; GKI, glucose-ketone index; H&E, haematoxylin and eosin.
